# Contrasting Function of Structured N-Terminal and Unstructured C-Terminal Segments of *Mycobacterium tuberculosis* PPE37 Protein

**DOI:** 10.1128/mBio.01712-17

**Published:** 2018-01-23

**Authors:** Javeed Ahmad, Aisha Farhana, Rita Pancsa, Simran Kaur Arora, Alagiri Srinivasan, Anil Kumar Tyagi, Madan Mohan Babu, Nasreen Zafar Ehtesham, Seyed Ehtesham Hasnain

**Affiliations:** a Inflammation Biology and Cell Signaling Laboratory, National Institute of Pathology, New Delhi, India; b Department of Biophysics, All India Institute of Medical Sciences, New Delhi, India; c Laboratory of Molecular and Cellular Biology, Centre for DNA Fingerprinting and Diagnostics, Hyderabad, India; d Medical Research Council, Laboratory of Molecular Biology, Cambridge, United Kingdom; e Department of Biochemistry, University of Delhi South Campus, New Delhi, India; f Molecular Infection and Functional Biology Laboratory, Kusuma School of Biological Sciences, Indian Institute of Technology, New Delhi, India; g Dr. Reddy’s Institute of Life Sciences, University of Hyderabad Campus, Hyderabad, India; h JH Institute of Molecular Medicine, Jamia Hamdard, Hamdard Nagar, New Delhi, India; Korea Advanced Institute of Science and Technology

**Keywords:** apoptosis, eukaryotic linear motifs, intrinsically disordered regions, molecular mimicry, nuclear localization signal, tolerogenic immune cells, host-pathogen interactions

## Abstract

Pathogens frequently employ eukaryotic linear motif (ELM)-rich intrinsically disordered proteins (IDPs) to perturb and hijack host cell networks for a productive infection. *Mycobacterium tuberculosis* has a relatively high percentage of IDPs in its proteome, the significance of which is not known. The *Mycobacterium*-specific PE-PPE protein family has several members with unusually high levels of structural disorder and disorder-promoting Ala/Gly residues. PPE37 protein, a member of this family, carries an N-terminal PPE domain capable of iron binding, two transmembrane domains, and a disordered C-terminal segment harboring ELMs and a eukaryotic nuclear localization signal (NLS). PPE37, expressed as a function of low iron stress, was cleaved by *M. tuberculosis* protease into N- and C-terminal segments. A recombinant N-terminal segment (P37N) caused proliferation and differentiation of monocytic THP-1 cells, into CD11c, DC-SIGN (dendritic cell-specific intercellular adhesion molecule-3-grabbing nonintegrin)-positive semimature dendritic cells exhibiting high interleukin-10 (IL-10) but negligible IL-12 and also low tumor necrosis factor alpha (TNF-α) secretion—an environment suitable for maintaining tolerogenic immune cells. The C-terminal segment entered the macrophage nucleus and induced caspase-3-dependent apoptosis of host cells. Mice immunized with recombinant PPE37FL and PPE37N evoked strong anti-inflammatory response, validating the *in vitro* immunostimulatory effect. Analysis of the IgG response of PPE37FL and PPE37N revealed significant immunoreactivities in different categories of TB patients, *viz.* pulmonary TB (PTB) and extrapulmonary TB (EPTB), vis-a-vis healthy controls. These results support the role of IDPs in performing contrasting activities to modulate the host processes, possibly through molecular mimicry and cross talk in two spatially distinct host environments which may likely aid *M. tuberculosis* survival and pathogenesis.

## IMPORTANCE

To hijack the human host cell machinery to enable survival inside macrophages, the pathogen *Mycobacterium tuberculosis* requires a repertoire of proteins that can mimic host protein function and modulate host cell machinery. Here, we have shown how a single protein can play multiple functions and hijack the host cell for the benefit of the pathogen. Full-length membrane-anchored PPE37 protein is cleaved into N- and C-terminal domains under iron-depleted conditions. The N-terminal domain facilitates the propathogen semimature tolerogenic state of dendritic cells, whereas the C-terminal segment is localized into host cell nucleus and induces apoptosis. The immune implications of these *in vitro* observations were assessed and validated in mice and also human TB patients. This study presents novel mechanistic insight adopted by *M. tuberculosis* to survive inside host cells.

## INTRODUCTION

Proteins are often presented as rigid bodies; they are in reality highly dynamic, which is important for their function and interactions. Moreover, certain proteins/protein fragments do not have a well-defined three-dimensional (3D) structure in solution, but assume such structures only in specific functional states (1). These, so-called, intrinsically disordered proteins (IDPs) have an amino acid composition that provides large structural flexibility and adaptability, enabling multiple interactions with diverse partners through short interaction motifs (2). Due to their advantageous properties, pathogens frequently employ disordered effector proteins (3) to manipulate the host machinery for their survival and dissemination, bypassing the host immune system. These disordered effectors hijack the host proteome through promiscuous interactions (4) involving molecular mimicry of host peptide motifs (5) in both viruses (6) and bacterial pathogens (4).

*Mycobacterium tuberculosis* exhibits a surprising capacity for adaptation to the host immune system. Predominantly resident in macrophages, it also survives within dendritic cells (DCs). These major antigen-presenting cells are required to initiate an immune response against intracellular pathogens and modulate the host immune response (7). Contrastingly, the semimature state of DCs, characterized by low costimulatory signals and lack of secretion of proinflammatory cytokines, is known to induce T-cell tolerance (8). Many pathogens modulate these functions by secreting proteins that mimic the activities of host cell factors (9). While inhibition of apoptosis is a well-known mycobacterial strategy to survive within the host macrophages, paradoxically, *M. tuberculosis* is known to deliberately induce apoptosis for its successful dissemination (10).

Although genome sequence data (11) and expression profiles of virulent, avirulent, and clinical *M. tuberculosis* strains have contributed to a better understanding of its infection biology, not much is known about the functions of the *Mycobacterium*-specific PE-PPE family proteins. This protein family constitutes ~10% of *M. tuberculosis* protein coding genes. These proteins are usually restricted to virulent mycobacterial species, and individual members of this protein family have been implicated in the disease process (12–14), generating antigenic variation, immune evasion, immune quorum sensing, cell death (15, 16), etc. Given the enrichment of interaction-prone disordered regions within the PE-PPE proteins, we hypothesized that they might directly modulate host cell functions (17). The family comprises three subfamilies (PE, PE-PGRS, and PPE) that include proteins with complex domain architectures (18) and extended disordered and/or low-complexity regions (19), the functional relevances of which are yet to be elucidated. Based on analysis of their sequence variation within mycobacteria, we selected a member of the PE-PPE family, PPE37, coded for by the *Rv2123* gene, for dissection of the functional implications of the presence of such disordered regions. Our results provide evidence supporting the role of intrinsically disordered stretches within a PPE protein in performing contrasting functions to modulate host processes, including immune regulation to favor the pathogen, through molecular mimicry and cross talk in two spatially distinct host environments.

## RESULTS

First we investigated the sequence variations of PE-PPE proteins with their closest homologues from 28 different strains of *M. tuberculosis* (see Table S1 in the supplemental material). These homologues were selected by construction of multiple sequence alignments using ClustalW (for selection criteria, see Materials and Methods). Although orthologs were missing, not annotated, or could not be identified in some strains based on our criteria, most identified orthologs showed very limited variations with no predictable functional relevance (e.g., single-residue changes in regions with no known functional relevance). On the other hand, when performing a similar variation analysis using the different species of *Mycobacterium* (see Table S2 in the supplemental material), we encountered too many missing orthologs and extended region losses/gains/variations to infer functional relevance to them. An interesting case was that of PPE37, which showed extensive sequence variations in some of the *M. tuberculosis* strains (e.g., F11 and 49-02) compared to the H_37_Rv sequence, but could still be undoubtedly identified in some of the nontuberculosis (non-TB) *Mycobacterium* species, with pronounced variations (indels of different sizes) falling into likely functional modules of the protein.

10.1128/mBio.01712-17.5 TABLE S1  *Mycobacterium tuberculosis* strains used for comparative analysis. Download TABLE S1, DOCX file, 0.1 MB. Copyright © 2018 Ahmad et al. 2018 Ahmad et al. This content is distributed under the terms of the Creative Commons Attribution 4.0 International license.

10.1128/mBio.01712-17.6 TABLE S2  *Mycobacterium* species used for comparative analysis. Download TABLE S2, DOCX file, 0.1 MB. Copyright © 2018 Ahmad et al. 2018 Ahmad et al. This content is distributed under the terms of the Creative Commons Attribution 4.0 International license.

**FIG 1:**
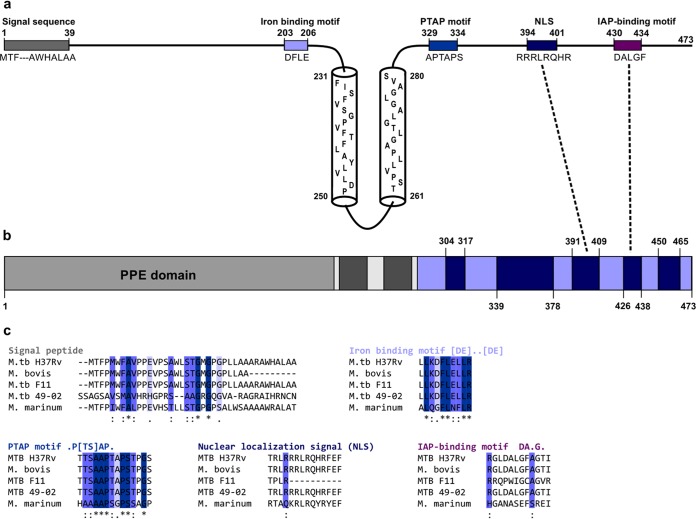
Computational analysis of PPE37. (a) A schematic representation of the PPE37 protein with transmembrane helices and potentially functional motifs indicates their residue boundaries and sequence. (b) A domain map of PPE37 with the PPE domain (light gray), transmembrane helices (dark gray), predicted disordered regions by IUPred (light blue), and ANCHOR-type disordered binding sites (dark blue with residue boundaries) is shown. The coincidence of two potentially functional linear motifs with ANCHOR-predicted disordered binding sites is highlighted by dashed lines. (c) The sequence regions of the predicted potentially functional motifs plus a few residue positions on both sides were cut from the Clustal Omega 1.2.3 alignment of 5 representative PPE37 homologues and are shown in the order of their occurrence within the PPE37 sequence (with motifs in the upper and bottom row corresponding to the N- and C-terminal fragments, respectively). The names and regular expressions of the aligned motifs are depicted in the same color as the corresponding box in the schematic representation of the protein. Below the aligned sequences, the Clustal consensus pattern provides information on their sequence conservation.“*” indicates positions with fully conserved residues (dark blue columns).“:” and “.” indicate if a strong or a weak amino acid group is fully conserved in the given position (middle and light blue columns, respectively). (The positive-scoring amino acid groups of the Gonnet Pam250 matrix were subdivided into “strong” [score of >0.5] and “weak” [score of ≤0.5] groups, respectively.) M.tb, *M. tuberculosis*.

Computational analyses of PPE37 sequence revealed an N-terminal PPE domain (225 amino acids [aa]; P37N]) (20) and a C-terminal intrinsically disordered region (191 aa; P37C) separated by two central transmembrane segments with a small helical hinge region in between (Fig. 1a and b). In the N-terminal part, a eukaryotic-type signal sequence spans residues 1 to 40 (21), probably driving periplasmic or extracellular localization, and an iron-binding motif is present close to the transmembrane anchor (22). The disordered C-terminal segment has a predicted nuclear localization signal (NLS) (23) and a few potentially functional predicted eukaryotic linear motifs (ELMs), such as a UEV domain-binding PTAP motif (usually mediating entrance to endosomes), and an IAP (inhibitors of apoptosis)-binding motif (IBM) capable of promoting apoptosis. These are relatively well-defined motifs that are not likely to occur by chance, and two of them even reside within ANCHOR-predicted disordered binding sites, which further underlines their likely functional importance (Fig. 1a and b).

The alignment of five representative PPE37 orthologs from different *M. tuberculosis* strains and mycobacterial species revealed remarkable sequence conservation of the predicted motifs (Fig. 1c). A striking feature was the absence of a stretch of 9 amino acids in the signal sequence of the otherwise 100% identical ortholog from the nontuberculous species *Mycobacterium bovis*. In the clinically prevalent H_37_Rv F11 strain, there are extensive variations within the disordered C-terminal segment that carries a stronger and more pronounced NLS extending from residues 402 to 425 (see Fig. S1a in the supplemental material), as predicted by PredictNLS. The presence of a stronger NLS in the more prevalent and virulent F11 strain and the conservation of the NLS sequence in an otherwise rapidly evolving disordered region (24) (Fig. S1a) suggest an important functional role for this eukaryotic motif within a bacterial protein. Although present in all other orthologs, apoptosis-promoting IBM motifs were not detected in the F11 counterpart, suggesting that it may follow another strategy for host modulation. Intriguingly, the PPE37 ortholog of the free-living, nontuberculous species *Mycobacterium marinum* lacked the iron-binding, PTAP, and NLS motifs.

10.1128/mBio.01712-17.1 FIG S1  Sequence similarities between PPE37 and other members of the PE-PPE family. (a) Multiple sequence alignment of representative orthologs of PPE37 by Clustal Omega with likely functional interaction motifs named and highlighted in different colors. The NCBI identifiers of the protein sequences are provided following the species names in the first line of the alignment. Motifs were searched by the ELM browser in each sequence and highlighted even if positionally not fitted with the corresponding H_37_Rv PPE37 motif. (b) Multiple sequence alignment of the five PPE37 paralogs by Clustal Omega with likely functional interaction motifs named and highlighted in different colors. Due to the poor conservation of sequences in certain regions, the motifs were searched by the ELM browser in each sequence and highlighted even if not fitted with the respective PPE37 motif. Three strongly conserved motif-like regions of unknown function, at the C-terminal region, are marked by blue rectangles. Clustal consensus patterns are indicated below the sequences as in Fig. 1c. (c) STRING analysis of PPE37 for protein-protein interacting partners, which include *hisE* (phosphoribosyl-ATP pyrophosphatase), *hisG* (ATP phosphoribosyltransferase), and the gene coding for hypothetical protein Rv2120c. Download FIG S1, DOCX file, 0.3 MB. Copyright © 2018 Ahmad et al. 2018 Ahmad et al. This content is distributed under the terms of the Creative Commons Attribution 4.0 International license.

Similarity search for PPE37 within the *M. tuberculosis* H_37_Rv proteome identified five close paralogs, namely, Rv0265c, Rv0453, Rv0096, Rv0286, and Rv3018c, all belonging to the PPE family. Previous reports on the evolution of PE and PPE proteins also indicated coclustering of these five genes as a subfamily affiliated with sublineage II PPW (proline-proline-tryptophan) of the PPE protein family and were shown to be upregulated in different intracellular microenvironments, perhaps performing similar functions (25, 26). While these paralogs show high sequence similarity within the region spanning the signal sequence and the PPE domain, many of them also harbor a potential iron-binding motif: the transmembrane regions and the disordered C-terminal segment show very low similarity, and with the exception of PPE2, the lack of PTAP motif and IBM patterns (Fig. S1b). Even so, almost all the paralogs harbor a positively charged patch of residues within the C-terminal region that could serve as an NLS, and they have three patches of strongly conserved positions toward their C termini that could not be identified as known motifs but most probably mediate important functions (Fig. S1b). It is important to note that PPE37 is not an essential protein for *M. tuberculosis* pathogenesis. It is, however, possible that these paralogs can functionally substitute for each other to some extent, and thus conclusions on their contribution to host invasion can only be drawn after knocking them out collectively.

**FIG 2:**
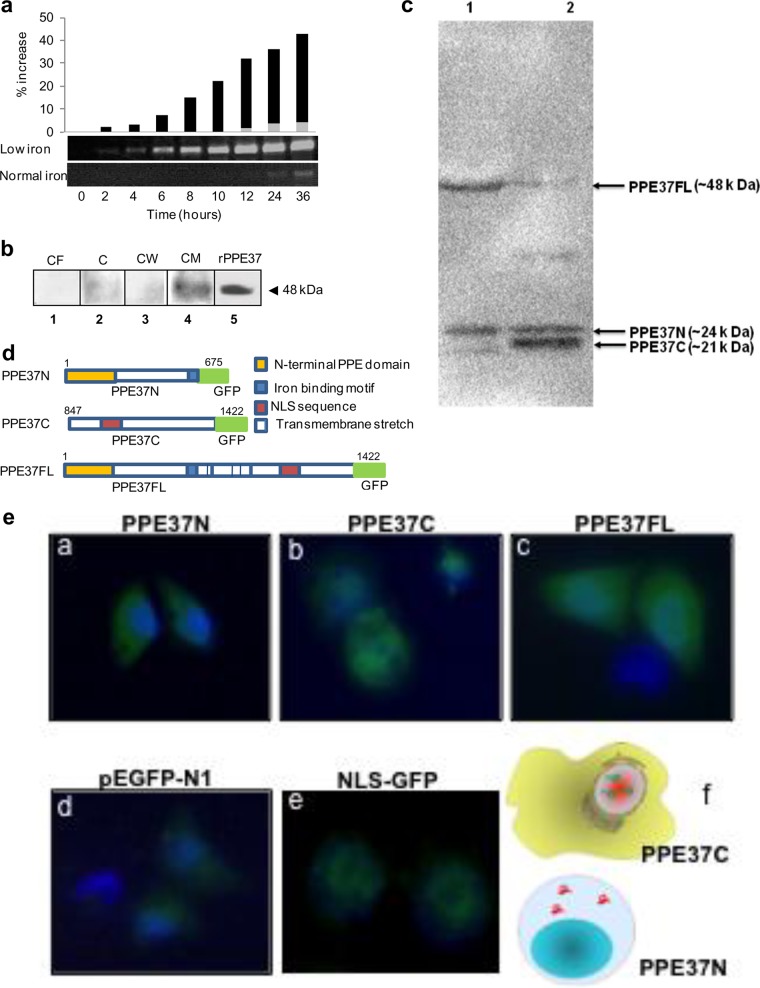
PPE37, induced under iron-deficient conditions, is present in the cell membrane and is cleaved into N- and C-terminal fragments that are localized to the cytoplasm and nucleus, respectively, of THP-1 cells. (a) PPE37 is induced under low-iron conditions. PPE37 gene expression was determined using real-time PCR at different time points. (b) PPE37 expressed under low-iron conditions is localized to the cell membrane. Immunoblot analysis of various cell fractions of *M. tuberculosis* H_37_Rv, grown under low-iron conditions, was carried out using anti-P37FL antibody. CF, culture filtrate; C, cytosol; CW, cell wall; CM, cell membrane; rPPE37, purified recombinant PPE37 protein used as a positive control. An arrowhead indicates the position of the 48-kDa protein band. Note the predominance of the 48-kDa PPE37 protein in the cell membrane fraction. (c) PPE37 is cleaved into N- and C-terminal segments by *M. tuberculosis* proteases. Immunoblot of PPE37FL protein expressed in *E. coli* BL21(DE3) using anti-His antibody. In lane 1, *E. coli* BL21(DE3) lysate was incubated with *M. tuberculosis* H_37_Rv lysate grown under normal-iron conditions, and lane 2 represents a blot of *E. coli* BL21(DE3) lysate when incubated with *M. tuberculosis* H_37_Rv lysate grown under depleted-iron conditions. (d) Schematic of different GFP fusion constructs used in transfection. Segments of PPE37 gene were fused to green fluorescent protein, and the different fusion constructs, namely, the full-length construct (PPE37FL), C-terminal fusion construct (PPE37C), and N-terminal fusion construct (PPE37N), were used to transfect THP-1 cells. The color codes are as follows: yellow, N-terminal region carrying signal sequence; blue, iron-binding motif; blue with vertical bars, transmembrane segments; red, NLS; green, GFP coding sequence. (e) Differential localization of N- and C-terminal fragments of PPE37. Shown is immunofluorescence analysis of the THP-1 cells transfected with various PPE37-GFP fusion constructs: PPE37N (panel a), PPE37C (panel b), PPE37FL (panel c), pEGFP-N1 vector control (panel d), and NLS (aa 394 to 401) fused to GFP (panel e). Nuclear staining with DAPI (4′,6-diamidino-2-phenylindole [blue]) and expression and localization of GFP (green) are shown. A schematic cartoon depicting differential localization of the 2 fragments of PPE37 is shown in panel f.

The presence of transmembrane stretches pointed to PPE37 likely sitting in the mycobacterial cell membrane. The *Rv2123* gene that encodes PPE37 is known to be under the IdeR regulator and induced by low-iron conditions, which was also confirmed by real-time PCR analyses at different time points (Fig. 2a). *M. tuberculosis* was cultured under low-iron conditions for 36 h, and immunoblot analysis of its various cell fractions, including the culture filtrate (CF), cytosol (C), cell wall (CW), and cell membrane (CM) fractions, using anti-P37FL (anti-PPE37 full-length) antibody indicated the presence of the 48-kDa PPE37 protein mainly in the CM fraction (Fig. 2b, lane 4), but not in the CF (lane 1) or CW (lane 3) fractions.

PPE37 is membrane anchored, but at the same time it has a number of motifs and NLS, which led us to suspect that a fragment of this protein might be secreted into the host cell cytosol upon proteolytic cleavage *in vivo*. To test this, *Escherichia coli* BL21(DE3) lysate overexpressing PPE37FL protein His tagged at both N- and C-terminal ends was prepared in lysis buffer as described in Materials and Methods. The cell lysate was incubated for 40 min at room temperature with *M. tuberculosis* H_37_Rv lysate grown under iron-depleted and normal-iron conditions. Western blotting was performed using anti-His antibody. The presence of cleavage products corresponding to ~24-kDa and ~21-kDa N-terminal and C-terminal protein segments, respectively, can be seen in lane 2, where iron-depleted *M. tuberculosis* H_37_Rv lysate was used (Fig. 2c, lane 2). Minimal cleavage was also observed when lysate of H_37_Rv was prepared from cells grown under normal-iron conditions. The presence of faint bands could be indicative of low expression of the protease required for proteolysis of PPE37 under normal-iron conditions.

The proteolysis of PPE37 into an active N-terminal iron-binding segment and an intrinsically disordered NLS-harboring C-terminal segment by protein factors exclusive to *M. tuberculosis* could be of functional importance *in vivo* during active mycobacterial growth. *In silico* predictions using genomic neighborhood and operon association employing the STRING database (27) showed the likely involvement of conserved integral membrane proteins Rv2120c (which harbors all the features of an S2P protease) and/or perhaps the already reported Rv2869c (28), in regulated intramembrane proteolysis (RIP) of the PPE37 protein (Fig. S1c).

The N-terminal segment was assessed for its iron binding capacity. Ferene-S staining of *M. tuberculosis* rP37FL (see Fig. S2a in the supplemental material) and atomic absorption spectroscopy of the N-terminal segment (rP37N) purified in iron-free forms (Fig. S2b) demonstrated their binding to ferric iron in a 1:1 molar ratio, which could be attributed to the presence of an iron-binding “Glu/Asp-X-X-Glu/Asp” motif (22). Furthermore, the comparison of the circular dichroism (CD) spectra of the unbound protein and protein samples incubated with various concentrations of FeCl_3_ demonstrated a concomitant decrease in the overall α-helical content of the protein (troughs at 210 and 222 nm) upon iron binding (Fig. S2c). Besides, the modest presence of random coil conformation in rP37N, typical of disordered regions, was also evident.

10.1128/mBio.01712-17.2 FIG S2  Recombinant PPE37 protein binds iron. (a) Ferrene-S staining of rPPE37 (lanes 1 and 2) was carried out in the absence (lane 1) or presence (lane 2) of iron. (b) Atomic absorption spectroscopy revealed the iron-binding property of PPE37 as a direct function of iron concentration. (c) Circular dichroism spectrum of rPPE37N (N-terminal segment) at different concentration of ferrous iron. (d) Immunoblotting confirms localization of the N-terminal and C-terminal regions to cytoplasm and nucleus, respectively. Cytoplasmic and nuclear lysates prepared from THP-1 cells transfected with pC-P37FL (lane 1), pC-P37N (lane 2), or pC-P37C (lane 3) were immunoblotted using anti-P37FL antibody. Download FIG S2, DOCX file, 0.1 MB. Copyright © 2018 Ahmad et al. 2018 Ahmad et al. This content is distributed under the terms of the Creative Commons Attribution 4.0 International license.

To check the localization of two segments within the host cells, green fluorescent protein (GFP)-tagged constructs of full-length *M. tuberculosis* PPE37 (PPE37FL; 1,422 bp), the N-terminal segment (PPE37N; aa 1 to 675) and the C-terminal segment (PPE37C, aa 847 to 1422) as shown schematically in (Fig. 2d) were transfected into THP-1 cells and subjected to immunofluorescence analysis 24 h after transfection. This revealed the cytoplasmic localization of the N-terminal segment, nuclear localization of the C-terminal segment, and predominantly cytoplasmic localization of the full-length protein (Fig. 2e, subpanels a to d). The vector pEGFP-N1 was taken as a control, wherein the fluorescence was primarily localized to the cytoplasm (subpanel d). The GFP-tagged NLS sequence (aa 394 to 401) of PPE37, N1-NLS, localized to the nucleus as expected (subpanel e). The PPE37C-transfected cells, however, started to die after 24 to 28 h of transfection, and therefore, the fluorescence was not monitored at later time points. Cells transfected with PPE37FL also underwent apoptotic cell death after 36 h of transfection, in a fashion similar to PPE37C. These results imply that the N-terminal segment localizes to the host cell cytoplasm, whereas the NLS-harboring disordered C-terminal segment is targeted to the nucleus and might interfere with host cell survival.

**FIG 3:**
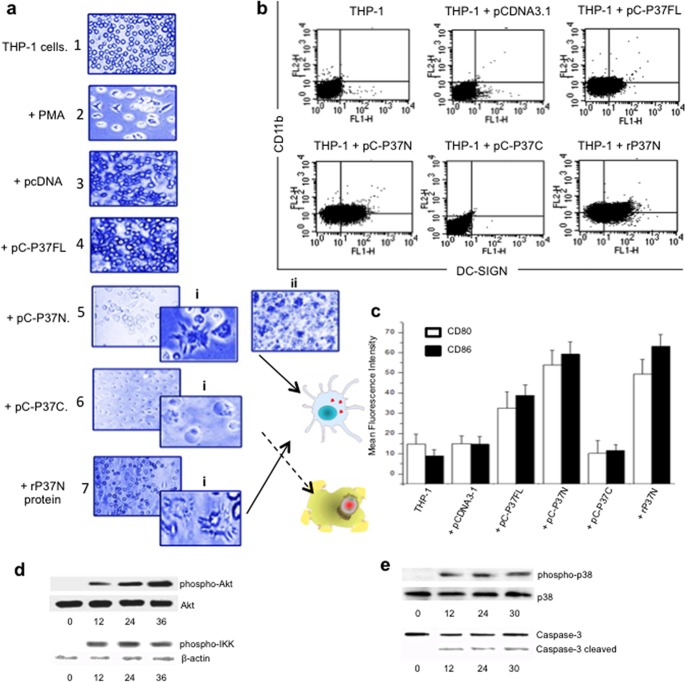
The N-terminal fragment of PPE37 induces cellular proliferation and differentiation and tolerance against *M. tuberculosis* antigens, whereas the C-terminal fragment causes cell death. (a) Differential fate of THP-1 cells transformed with different PPE37 constructs. Shown are results from morphological analysis of THP-1 cells (panel 1), those after differentiation with phorbol myristate acetate (PMA [panel 2]), THP-1 cells transfected with pCDNA 3.1 (panel 3), pC-P37FL (panel 4), pC-P37N (panel 5), or pC-P37C (panel 6) or THP-1 cells incubated with purified rP37N (3 μg) (panel 7). pC-P37N-transfected THP-1 cells observed at lower resolution show a higher number of cells (subpanel 5ii). Subpanel 5i depicts higher-magnification images of cells seen, subpanel 6i shows apoptotic cells with cellular blebbing, and differentiated THP-1 cells after incubation with rP37N can be seen in subpanel 7i. The fates of cells carrying N-terminal (top) and C-terminal (bottom) fragments of PPE37 are depicted in a cartoon to the right of panels 6 and 7. These images were taken at 20× resolution, whereas images in subpanels 5i, 6i, and 7i were taken at 40× resolution. (b) THP-1 cells transfected with PPE37 N-terminal fragment display increased surface expression of dendritic cell markers. Shown are results from immunophenotyping analysis of THP-1 cells or cells transfected with pCDNA3.1, full-length pC-PPE37FL, the N-terminal segment of pC-PPE37N, or the C-terminal end of pC-PPE37C. Note the increased expression of dendritic cell markers in THP-1 cells either transfected with the pC-PPE37N construct or treated with purified rP37N protein. (c) Surface expression of other dendritic cell markers like CD80 and CD86 is also enhanced in THP-1 cells treated with purified rP37N protein or transfected with pC-PPE37N. THP-1 cells were transfected with vector alone or pC-PPE37FL, PPE37N, or pPE37C or incubated with purified PPE37 protein and assayed for expression of CD80 and CD86. THP-1 cells matured with GM-CSF (100 ng/ml), IL-4 (200 ng/ml), and TNF-α (20 ng/ml) were taken as a mature DC control. (d) P37C-dependent cell death follows the caspase activation pathway. Shown are results from immunoblotting of the nuclear extract of THP-1 cells incubated with rP37N protein and probed with antibodies to phospho-Akt, total Akt, and phospho-IKKα at 0, 12, 24, and 36 h postincubation. β-Actin was used as a loading control. Transfection was carried out with 6 μg/ml of each construct, and incubation with rP37N was carried out with 3 μg/ml of recombinant protein in all cases. (e) Immunoblotting of whole-cell lysates of THP-1 cells transfected with pC-P37C at 0, 12, 24, and 30 h posttransfection results in the increased phosphorylation of p38 and activation of caspase-3 leading to a cleaved product at 12, 24, and 30 h of transfection.

To evaluate the functional consequence of the differential localization of N- and C-terminal segments of PPE37 within the host cell, morphological analysis of THP-1 cells was carried out after transfection with pC-P37FL, pC-P37N, and pC-P37C. In these experiments, pcDNA3.1 was used as a vector backbone instead of pEGFP-N1 to rule out any possible interference of the GFP tag. The expression and cellular localization of the resultant proteins after transfection were first ascertained by immunoblotting of the nuclear and cytoplasmic lysates. The presence of the N-terminal segment in the cytoplasmic fraction (Fig. S2d, upper panel, lane 2) and the C-terminal segment in the nuclear fraction (Fig. S2d, lower panel, lane 3) was evident. Significant cytoplasmic localization (Fig. S2d, upper panel, lane 1) and a faint nuclear localization of PC-P37FL (lower panel, lane 1) could also be seen. Phase-contrast microscopy was then carried out to assess the effect of the N- and C-terminal segments on the overall morphology of the monocytic THP-1 cell line. After 24 h of transfection with pC-P37N (Fig. 3a, panel 5) or incubation with purified rP37N protein (Fig. 3a, panel 7), THP-1 cells were seen to undergo proliferation and differentiation (Fig. 3a, panels 5 and 7, subpanels 5i and 7i) into adherent stellate cells with dendritic cell-like morphology. These cells also divided at a higher than normal rate compared to the untransfected THP-1 cells (Fig. 3a, compare subpanels 5ii and 7i with panel 1) or cells transfected with control vector (Fig. 3a, compare, subpanels 5ii and 7i with panel 1), and a visible increase in the number of cells could be seen (Fig. 3a, subpanel 5ii). In contrast, cells transfected with pC-P37C (Fig. 3a, panel 6), earlier shown to localize to the THP-1 nucleus (Fig. 2e, panel b), were seen to undergo apoptotic cell death, evident from blebbing and spilling of the cellular components (Fig. 3a, panel 6, subpanel 6i). The observed apoptotic cell death could well be induced by the conserved proapoptotic IAP (inhibitors of apoptosis)-binding motif (IBM) present between residues 430 and 433 of PPE37 (Fig. 1), possibly through binding to IAP proteins, thereby leading to activation of caspases resulting in apoptosis. At this time, this mechanistic explanation, however, remains a speculation in the absence of experimental evidence and support thereof. The expression of full-length construct pC-P37FL, however, did not lead to any significant morphological sign of cellular differentiation or apoptosis after 24 h of transfection (Fig. 3a, panel 4). The untransfected THP-1 cells and control vector-transfected cells (Fig. 3a, panels 1 and 3) grew normally. Transfection of RAW264.7 or J774 cells (instead of THP-1 cells) with pC-P37C as well as pC-P37FL also corroborated the same results (data not shown).

Transfected THP-1 cells were further subjected to cell cycle analysis to evaluate the proliferation and apoptosis induced by the N- and C-terminal segments of PPE37, respectively. THP-1 cells, after 24 h of transfection with pC-P37N, showed a higher percentage of cells in S phase, indicative of proliferation (see Fig. S3a, panel 4, in the supplemental material). In contrast, pC-P37C-transfected cells exhibit apoptosis, as evident from the higher percentage of cells in G_0_ phase (Fig. S3a, panel 5). On the other hand, THP-1 cells transfected with pC-P37FL also showed significantly higher numbers of cells in the G_1_ and S phases compared to the controls (Fig. S3a, panel 3). Cell cycle analysis of THP-1 cells incubated with rP37N protein in iron-quenched medium was also carried out to determine the potential of rP37N in cell proliferation. THP-1 cells analyzed for cell cycle at the start of the experiment (*t* = 0 after addition of 2′2′-dipyridyl [DP]) were taken as the control (Fig. S3b, panel 6). It could be seen that under iron depletion, the presence of rP37N leads to proliferation (Fig. S3b, panel 7) and subsequent differentiation, while the absence of rP37N results in increased cell death (Fig. S3b, panel 8). These results demonstrate that the N-terminal segment of P37N modulates cell proliferation, whereas the C-terminal segment enhances apoptosis, complementing earlier results based on morphological analysis using light microscopy.

10.1128/mBio.01712-17.3 FIG S3  Cell cycle analyses of THP-1 cells transfected with various constructs. (a) Histograms indicating the percentage of cells in each cell cycle phase for THP-1 cells, THP-1 cells transfected with pcDNA 3.1, pC-P37FL, pC-P37N, or pC-P37C after 24 h of transfection. pC-P37N-transfected cells (panel 4) showed an increase in the S phases, whereas pC-P37C-transfected cells (panel 5) showed higher G_0_ phase indicating cell death. The quantitative estimation of cells at different stages of the cell cycle is shown in the table below panel b. (b) Cell cycle profile of THP-1 cells (panel 6) at the start of 2′-2′-dipyridyl treatment (*t* = 0 h [panel 6]), after 6 h of treatment followed by 18 h of recovery in RPMI (*t* = 24 h [panel 7]), and after incubation with rP37N in the presence of 2′-2′-dipyridyl with treatment carried out for 6 h followed by recovery in RPMI (*t* = 24 h [panel 8]). Download FIG S3, DOCX file, 4.1 MB. Copyright © 2018 Ahmad et al. 2018 Ahmad et al. This content is distributed under the terms of the Creative Commons Attribution 4.0 International license.

To further investigate the effects of the secreted N-terminal segment of PPE37 on the proliferation and differentiation of myeloid cells, the immunophenotype of untransfected THP-1 cells or those transfected with pC-P37FL, pC-P37N, or pCP37C or incubated with rP37N protein was evaluated by assessing the overall surface expression of DC differentiation markers through detection of indirect immunofluorescence using a flow cytometer. The expression of DC differentiation determinants CD11c and DC-SIGN (dendritic cell-specific intercellular adhesion molecule-3-grabbing nonintegrin) on THP-1 cells transfected with pC-P37N or incubated with rP37N is evident from double-positive cells and is comparable to the granulocyte-macrophage colony-stimulating factor (GM-CSF), interleukin-4 (IL-4), and tumor necrosis factor alpha (TNF-α) differentiated DCs. Additionally, these cells also displayed an intermediary increase in CD80 and CD86 (Fig. 3c) surface markers. However, cytokine profiling showed high IL-10 and negligible IL-12 and TNF-α secretion profiles (data not shown). This repertoire of surface markers and cytokine profiles defines the semimature (intermediate) stage of DC (smDC) differentiation and is known to be instrumental in inducing immune tolerance against the allogeneic antigen.

Having shown that rP37N stimulates the differentiation of monocytic cells to smDCs, we sought to delineate the signaling cascade involved in the process. NF-κB being an important pathway, we analyzed molecular effectors upstream of NF-kB by immunoblotting at different time points which indicated a time-dependent increase in the phosphorylated form of Akt (Fig. 3d, upper panel) and a sustained presence of phospho-IKK up to 36 h after transfection (Fig. 3d, lower panel). These results suggest that P37N-mediated proliferation and differentiation of THP-1 cells and subsequent surface expression of differentiation markers (Fig. 3b and c) involve Akt and NF-κB signaling pathways. However, the precise signaling processes leading to differentiation and proliferation remain to be explored.

Cells transfected with pC-P37C undergo apoptotic cell death, as could be seen from morphological (Fig. 3a) and cell cycle (Fig. S3a and b) analyses. We further sought to analyze the cellular effectors that modulate this outcome. Immunoblotting of the THP-1 cell lysates at 12, 24, and 36 h of transfection with pC-P37C demonstrated the activation of caspase-3, as evident from the presence of low-molecular-mass cleavage products (Fig. 3e). The apoptotic nature of pC-P37C is also validated through transfection studies in HEK293T cells. These cells were transfected with pC-PPE37FL, pC-PPE37N, pC-PPE37C, and control vector pcDNA3.1(+). The apoptotic assay was done after 24 h through flow cytometry (see Fig. S4 in the supplemental material). Cells transfected with pC-PPE37C showed the highest percentage of apoptotic cells compared to the control (Fig. S4d and f). However, when transfection of pC-PPE37C was carried out in the presence of apoptotic inhibitor, an inhibition of apoptosis was seen (Fig. S4e and f). These results corroborated our previous results, which depicted the apoptotic nature of the C-terminal segment of PPE37 (Fig. 3a).

10.1128/mBio.01712-17.4 FIG S4  Apoptosis assay in the presence of apoptotic inhibitor. pcDNA constructs of PPE37FL, PPE37N, and PPE37C were transfected in HEK293T cells, and apoptosis analysis was done using a flow cytometric procedure. Representative FACS plots are shown in panels a to e. (a) pcDNA3.1(+) control. (b) pC-PPE37FL. (c) pC-PPE37N. (d) pC-PPE37C. (e) pC-PPE37C plus inhibitor. In panel f, the number of apoptotic cells is represented by a bar diagram. Download FIG S4, DOCX file, 0.5 MB. Copyright © 2018 Ahmad et al. 2018 Ahmad et al. This content is distributed under the terms of the Creative Commons Attribution 4.0 International license.

The analysis of cytokine secretion by pC-P37C-transfected THP-1 cells monitored from the start of transfection for 36 h showed a time-dependent increase in the secretion of IL-12p40 (which reaches maximum levels of 282 pg/ml at 36 h) and TNF-α (2,200 pg/ml) that is significantly higher than with the vector control. This was concomitant with a significantly reduced secretion of IL-10. Although cytokine secretion was monitored until 36 h of transfection with pC-P37C, the concentrations after 24 h may reflect the concentration of secreted as well as intracellular cytokine as the cells begin to undergo apoptosis after 24 h. The results, nonetheless, signify that pC-P37C-induced host cell death is either instrumental in enhanced secretion of IL-12p40 and TNF-α, or the upregulation of these two cytokines by P37C might be a factor in inducing apoptosis of the pC-P37C-transfected THP-1 cells.

**FIG 4:**
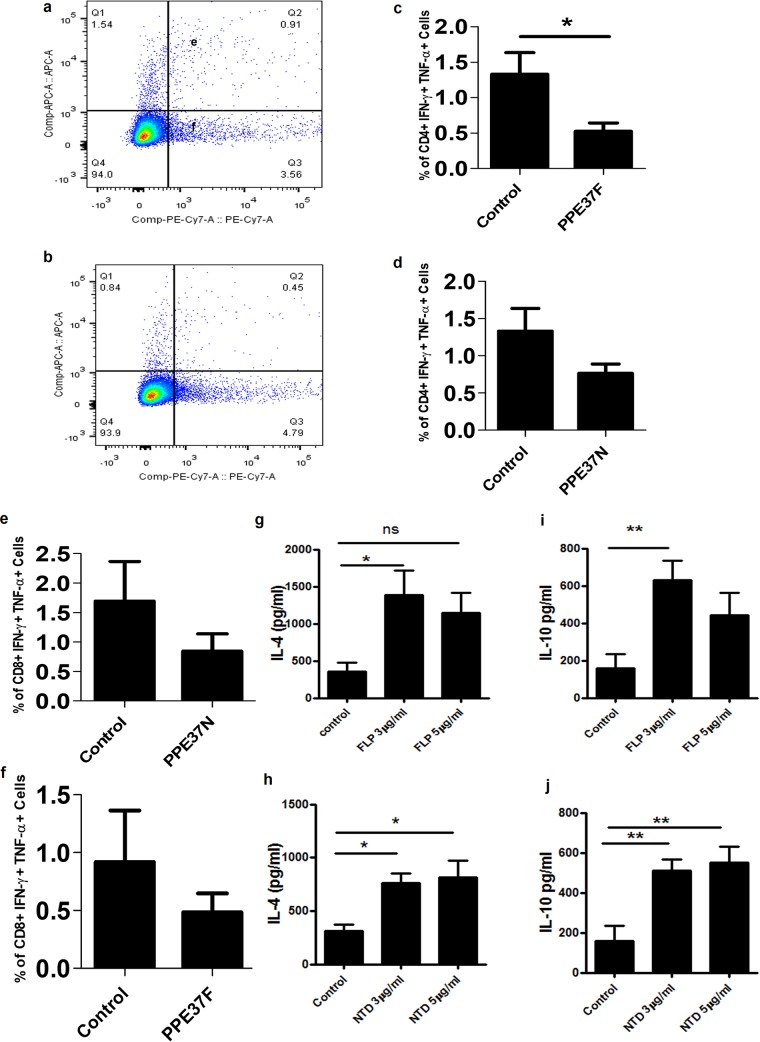
Recombinant purified PPE37FL and PPE37N proteins evoke anti-inflammatory response in immunized mice. (a and b) Representative fluorescence-activated cell sorter (FACS) plots of control and PPE37FL protein-treated cells. (c) Decrease in the number of CD4^+^ cells double positive for IFN-γ and TNF-α treated with rPPE37FL. (d) There is also a decrease in the number of CD8^+^ cells double positive for IFN-γ and TNF-α treated with rPPE37FL. (e and f) Decrease in the number of CD4^+^ and CD8^+^ polyfunctional cells double positive for TNF-α and IFN-γ compared to the control. (g) Significant increase in the level of IL-4 and decrease in secretion levels with increase in protein concentration of rPPE37FL (FLP). (h) The level of IL-4 increases in a dose-dependent manner. NTD, N-terminal domain. (i) The level of IL-10 was also found to increase in the case of rPPE37FL-treated cells and to follow a similar pattern to IL-4. (j) The IL-4 concentration increases with increasing concentration of rPPE37N protein antigen. A *P* value of <0.05 was considered significant. ns, not significant *, *P* ≤ 0.05; **, *P* ≤ 0.01.

The immunologic implications of the *in vitro* results described so far were functionally confirmed by evaluating the immunogenic properties of full-length PPE37 and the N-terminal segment in mice and also in human TB patients. Splenocytes isolated from mice immunized with recombinant purified PPE37FL and PPE37N proteins were restimulated with different concentrations of recombinant PPE37FL (rPPE37FL) and rPPE37N antigenic proteins as described in Materials and Methods. After 36 h, cells were harvested. Intracellular cytokine analysis using flow cytometry demonstrated a significant decrease in the number of CD4^+^ polyfunctional gamma interferon (IFN-γ)/TNF-α cells in comparison to the control in rPPE37FL-treated cells (Fig. 4a to c). A decrease can be seen in polyfunctional CD8^+^ cells treated with rPPE37FL protein, although the decrease is not statistically significant (Fig. 4d). However, in case of recombinant PPE37N, although a decrease in number of polyfunctional CD4^+^ and CD8^+^ IFN-γ/TNF-α cells was observed, this was not statistically significant (Fig. 4e and f).

We next evaluated the level of anti-inflammatory cytokines like IL-10 and IL-4 from restimulated splenocytes. Cytokine profiling after 48 and 96 h posttreatment showed enhanced secretion of anti-inflammatory cytokines like IL-10 and IL-4. rPPE37FL protein (3 μg/ml) elicited secretion of IL-10 and IL-4, whereas 5 μg/ml rPPE37FL did not show significant increase in IL-10 and IL-4 (Fig. 4g and i). However, rPPE37N (3 and 5 μg/ml) showed significantly increased secretion of both cytokines (Fig. 4h and j).

**FIG 5:**
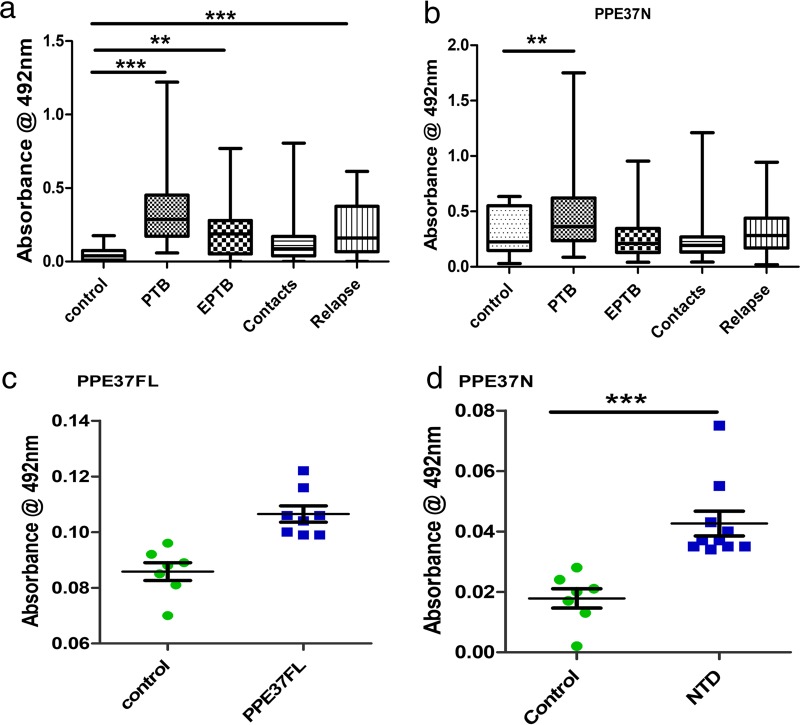
Recombinant PPE37FL and PPE37N protein antigens displayed strong B-cell response in tuberculosis patients and immunized mice. Immunoreactivity of sera from different categories of TB patients with rPPE37FL and rPPE37N was studied using ELISA. (a) The IgG response against PPE37FL shows statistical significance against PTB, EPTB, and relapse cases of tuberculosis, whereas with contacts, no such statistically significant response was seen. (b) The B-cell response against PPE37N reveals significance only with PTB cases, and no such significant immune reactivity was seen in serums of other categories. (c and d) B-cell responses from immunized mouse sera. Serum collected from blood of immunized mice was used for ELISA to determine the level of total IgG against respective proteins. In immunized mouse sera, the level of IgG against rPPE37FL was significant (c), and the level of IgG against rPPE37N was also found to be highly significant (d).

To evaluate the antigenic potential of recombinant proteins rPPE37FL and rPPE37N, experiments were designed to compare the IgG humoral response in different categories of TB patients like pulmonary tuberculosis (PTB), extrapulmonary tuberculosis (EPTB), TB patient contacts, and relapse cases of tuberculosis and compared with healthy controls. rPPE37FL showed a significant response in PTB, EPTB, and relapse cases compared to control. However, rPPE37N showed statistically significant immune response only with PTB cases compared to the control (Fig. 5b). We also analyzed the humoral immune responses of PPE37FL and PPE37N from sera of immunized mice and compared them with the responses of sera from the control group of mice. Our results showed a highly significant level of IgG humoral response against both antigens in comparison to control mice (Fig. 5c and d). Taken together, the data from mice and human TB patients functionally corroborate the *in vitro*-derived data in terms of immune regulatory properties likely to favor the pathogen.

## DISCUSSION

Identification and characterization of functionally important intrinsically disordered regions in the proteome will enable better understanding of infection biology and pathogenesis of pathogens like *M. tuberculosis* that survive within a range of microenvironments and have evolved a sophisticated repertoire of proteins and mechanisms to subvert host responses. Viruses use host-like peptide motifs to mediate successful infection (29), but whether a similar strategy is prevalent in *M. tuberculosis* or not remains to be investigated. In this study, we showed that disordered regions of a member of the *Mycobacterium*-specific PE-PPE protein family have important roles in pathogenesis. PPE37, induced under low-iron conditions during the course of infection, interferes with the evolutionarily conserved host pathways that include cell proliferation, differentiation, and apoptosis and is also required, along with other mycobacterial proteins, for causing a shift from a metabolically dormant form to an actively growing state, thereby providing strong evidence of its importance in virulence and disease progression. Moreover, antigenic protein PPE37 also modulates the immune system and elicits a propathogen response in immunized mice.

The results presented here provide a degree of mechanistic insight into the function of PPE37 that involves interplay between its N- and C-terminal segments. Induction of membrane-localized PPE37 during iron stress is speculated to act under the quorum sensing mechanism wherein a low iron concentration may indicate nutrient depletion resulting from an increase in bacterial load. Induction of PPE37 upon iron stress would be a prelude to its proteolytic cleavage to generate the N- and C-terminal segments and enable their subsequent extracellular or nuclear localization, respectively. After translocation to the host cell nucleus, the C-terminal segment induces apoptosis. Due to its predicted disordered nature, this segment must be structurally highly adaptable. This, together with the presence of the eukaryotic NLS and likely functional IBMs, argues for a mechanism that would allow the protein to mimic host protein(s), thereby interfering with the host machinery.

The observed caspase-3-dependent apoptosis of host cells, which is associated with the nuclear localization of the disordered C-terminal segment and might be directly induced by the conserved IBMs, may be a possible mechanism for *M. tuberculosis* propagation within the protected environment of apoptotic vesicles. It is interesting to note that the secreted SopB of *Salmonella enterica* serovar Typhimurium was shown to diversify its function by localizing to different host subcellular compartments in a ubiquitin-dependent manner, resulting in altered host vesicle trafficking (30). Thus, it appears that broadening the functional repertoire of a single protein by posttranslational modification leading to multiple outcomes could be common to several intracellular pathogens.

Moreover, hijacking host machinery appears to be facilitated by exposing short eukaryotic linear peptide motifs in intrinsically disordered segments of the polypeptide chain. Inhibition of apoptosis by mycobacteria helps in facilitating prolonged host survival and therefore safeguarding its persistence and replication within the host. In contrast, the host would like to induce apoptosis to clear off the infection (31). However, bacteria tend to exploit the anti-inflammatory properties of apoptosis, which will be advantageous for their systemic dissemination (32), and therefore mycobacteria would likely prevent apoptosis in the early phase of infection to allow their replication within the conducive, nutrient-rich milieu of the host. In the later phase, given the need for their dissemination due to nutritional scarcity as an important trigger factor, they induce cell death.

On the other hand, in the *in vivo* context, it seems logical that the proteolytically cleaved N-terminal segment with its plausible extracellular translocation, mediated by the presence of signal sequence, would be taken up by circulating monocytes leading to proliferation and differentiation into smDCs. Although stimulation of host immune cells by bacterial proteins has been widely studied, it has been reported recently that some bacterial proteins facilitate their uptake by host cells and interfere with signaling pathways (33) or induce selective expansion or suppression of host immune active cells (34). Although DCs play a key role in activating and expanding responses against pathogens in their mature states, the semimature cells thriving in a high-IL-10, low-TNF-α environment facilitate the development of T-cell tolerance (35).

The other key mechanism employed by *M. tuberculosis* for successful invasion and infection of host cells is also by disturbing the cell-mediated and humoral immune response balance. A number of proteins belonging to the PE-PPE family have been found to be involved in reducing the proinflammatory cytokines and enhancing secretion of propathogen anti-inflammatory cytokines (35). Cellular immune responses controlled by CD4^+^ and CD8^+^ T cells form the central element of adaptive immune response against *M. tuberculosis* (36). CD4^+^ cells are important during the acute phase of infection, whereas CD8^+^ has a role in clearance of infection during the chronic phase of infection. The suppression of proinflammatory cytokines like IFN-γ and TNF-α in immunized mice by decreasing the number of polyfunctional CD4^+^ and CD8^+^ T cells clearly depicts the propathogen immunomodulatory effect of the antigenic protein PPE37. The concentration-dependent decreasing effect of rPPE37FL on secretion of anti-inflammatory cytokine could possibly be due to the presence of the apoptotic C-terminal segment present in rPPE37FL protein.

**FIG 6:**
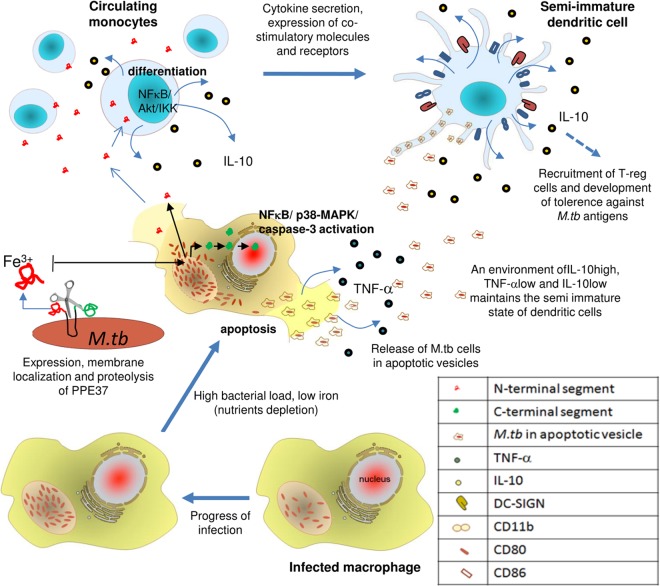
Proposed model for PPE37-mediated molecular mimicry and modulation of host cell processes. The high expression of PPE37 under low-iron conditions and subsequent proteolysis stimulate its differential localization to the host cells. The extracellular localization of the N-terminal segment and entry into the circulating monocytic cells lead to their differentiation and proliferation into semimature dendritic cells. On the other hand, the nuclear localization of the C-terminal end leads to host cell apoptosis.

Based on our observations, we hypothesize (Fig. 6) that mycobacteria might safeguard their dissemination as well as their future niche by the modulation and interplay of the two most crucial host mechanisms—apoptosis and cellular differentiation. The surface expression of DC-SIGN on semimature DCs would help bacterial uptake from neighboring apoptotic cells, where apoptosis is induced by the C-terminal segment that was cleaved and translocated to the nucleus as a result of critical iron depletion due to high bacterial load. This altruistic strategy of *M. tuberculosis* to propel the differentiation of peripheral monocytes into DC-SIGN-positive smDCs could provide a rich ground for HIV infection, complemented by depleting the population of potential immune active cells thereby causing an overall lowering of immunity against the pathogen. Results from experiments carried out in a mouse model and studies involving human TB patients reinforce the *in vitro* data supporting the likely immune regulation aided by this protein in favor of the pathogen. This therefore frames an elegant niche for bacterial propagation *in vivo* and opens up questions to investigate the role of PPE37 protein in intracellular persistence as well as dissemination of *M. tuberculosis* in infection models.

The findings presented here have important implications. While they provide and suggest novel targets for developing intervention strategies to inhibit pathogenesis (37), these also open up the possibility to further investigate and identify minimal peptides in the N- and C-terminal segments that can bring about antagonistic responses such as apoptosis and proliferation. Being able to do this would not only allow a better understanding of the molecular mechanisms, but would also provide us with a tool to trigger differentiation of immune cells under *in vitro* conditions and to investigate dynamics of cell populations. In conclusion, we demonstrate the potential of eliciting antagonistic outcomes in a host by the same bacterial protein upon posttranslational modification through proteolysis. We suggest that molecular mimicry, hijacking of host factors, and modulation of host cell survival, intrinsically facilitated by the disordered protein domains, may represent a paradigm shift in understanding tuberculosis biology and could be a generic mechanism adopted by other intracellular pathogens.

## MATERIALS AND METHODS

### Data set assembly and comparative analysis of PE-PPE proteins.

The proteome of *Mycobacterium tuberculosis* H_37_Rv was downloaded from NCBI (https://www.ncbi.nlm.nih.gov/). The PE-PPE proteins (see Table S3 in the supplemental material) were selected from the proteome and complemented by a few missing proteins from the Tuberculist database (http://tuberculist.epfl.ch/), namely, PE10, PE11, PPE40, PE_PGRS63, and PE_PGRS58. Also, the proteomes of other 28 *Mycobacterium tuberculosis* strains whose genome assembly and annotation were indicated as complete in NCBI (Table S1) were obtained for comparative analysis. For each H_37_Rv PE-PPE protein, the closest homologues were selected from the obtained strains using BLASTP. Those sequences were accepted as true orthologs which had a minimum of 50% mutual sequence coverage with the respective H_37_Rv query protein and that had also pulled the respective H_37_Rv query protein as the closest homologue when blasted against the H_37_Rv proteome.

10.1128/mBio.01712-17.7 TABLE S3  Protein families and secretome used in analysis. Download TABLE S3, DOCX file, 0.1 MB. Copyright © 2018 Ahmad et al. 2018 Ahmad et al. This content is distributed under the terms of the Creative Commons Attribution 4.0 International license.

### Comparative analysis of PPE37 homologues.

The PPE37 orthologs from different strains and also species of *Mycobacterium* were merged, and 5 representative sequences were selected for constructing an ortholog alignment using Clustal Omega 1.2.3. Among the selected sequences, the H_37_Rv sequence represented identical or highly similar sequences from 9 strains, and the MTB F11 sequence represented identical sequences from 5 strains, while the MTB 49-02 sequence formed a unique cluster on its own. The *M. bovis* sequence represented itself and the identical *M. bovis* BCG sequence. The *M. marinum* sequence formed a different cluster on its own.

### *In silico* analyses of PPE37 structure and interaction potential.

Disordered regions and disordered binding sites were predicted by ANCHOR^3^, which incorporates the predictions of the IUPred method (38). Residues were considered disordered if they scored >0.5 by IUPred. Annotated ELM patterns from ELM browser (http://elm.eu.org/) (39) that are relevant for humans were also searched in PPE37 using a dedicated function of ANCHOR; we restricted our analysis to low-probability motifs (ELM pattern probability of <0.001) within disordered regions to reduce false-positive hits. ELMs were accepted as disordered if any of their residues scored >0.4 by IUPred (40). The iron-binding motif (21) and the NLS (22) were detected based on the respective methods.

### Cell fractionation.

Two 100-ml cultures of *M. tuberculosis* H_37_Rv, grown in 7H9 medium until mid-log phase (optical density at 600 nm [OD_600_] of 1.0), were divided into two sets of 50 ml each and grown in either the absence (control, iron replete) or presence (iron depleted) of 100 μM 2′-2′ dipyridyl (DP) for another 24 and 36 h. These were fractionated to yield cell wall, cell membrane, and cytosolic fractions as described previously (31). Culture filtrate proteins were extracted, and PPE37 was detected by anti-PPE37 raised in mouse.

### PPE37 protein cleavage is mediated by *M. tuberculosis* proteases.

To ascertain proteolytic lysis of PPE37 protein into N- and C-terminal fragments, *Escherichia coli* BL21(DE3) cells were transformed with the pET28a-PPE37FL construct. Recombinant PPE37FL protein was induced by 1 mM IPTG (isopropyl-β-d-thiogalactopyranoside) at an OD of 0.6 for 3 h. Cells were centrifuged and washed once with phosphate-buffered saline (PBS). Cell lysis was carried out in lysis buffer (50 mM sodium phosphate buffer, 200 mM NaCl [pH 7.9]) through sonication. Similarly, *M. tuberculosis* H_37_Rv was grown in 7H9 medium until it reached an OD of 1, and then to chelate iron, 200 µM 2′-2′-dipydridyl (iron chelator) was added for 40 h. *M. tuberculosis* H_37_Rv cells grown under iron-depleted and iron-replete conditions were pelleted, and cell lysate was prepared in lysis buffer using a bead beater.

### Iron binding assays.

The iron-binding property of the N-terminal segment of PPE37 (rP37N) was assessed by atomic absorption spectroscopy using FeCl_3_ as a standard. Ferene-S staining, tryptophan fluorescence quenching, and the molar ratio of Fe^3+^ binding to rP37N were calculated by atomic absorption spectroscopy and analyzed with K2D software.

### Cell culture, infection, transfection, and protein incubation and stimulation.

THP-1 and J774 cells (National Centre for Cell Science, Pune, India) were cultured in RPMI 1640 and Dulbecco’s minimal essential medium (DMEM; Invitrogen, Grand Island, NY), respectively, with 10% fetal calf serum (FCS) and antibiotics. Infection with *M. tuberculosis* at a multiplicity of infection (MOI) of 1:10 was carried out followed by induction of iron stress.

Transfection plasmids containing the full-length N- or C-terminal segment of PPE37 were constructed using the pEGFP-N1 (Promega) plasmid backbone for GFP-tagged expression and separately in pcDNA 3.1 vector. Figure 2d depicts a schematic representation of different constructs generated for this study.

rP37N proteins, dialyzed against 50 µM DP, were initially treated with polymyxin B (Promega) to remove lipopolysaccharide (LPS) or endotoxin contamination, if any, and further evaluated by E-toxate gel formation analysis (Sigma). The purified proteins were then added (3 µg/ml) to about 60% confluent THP-1 culture. Incubation was carried out for periods of 2 and 12 h. The experiment was carried out in 60-mm dishes for cell cycle analysis, 4-chamber slides for immunofluorescence studies, and 96 well plates for enzyme immunoassay (EIA).

### THP-1 differentiation to mature DCs.

Differentiation and maturation of THP-1 cells into mature dendritic cells were carried out as previously described (32).

### Cell and nuclear extract preparation, cytokine assay, and flow cytometry.

Whole-cell extracts and cytoplasmic and nuclear extracts for immunoblot analysis were prepared as described earlier (16). THP-1 cells were seeded at a density of 2 × 10^5^ cells per well in a 24-well plate. Transfection with each construct was carried out as mentioned earlier. After 0, 12, 24, and 36 h of transfection or incubation with rP37N protein, the concentrations of secreted IL-10, IL-12p40, and TNF-α were monitored as per the manufacturer’s protocols for EIA. Cell cycle analyses of THP-1 cells, either transfected or incubated with rP37N, were carried out using flow cytometry after propidium iodide (PI) staining as described elsewhere (37). Immunophenotype analysis of the transfected or rP37N-incubated THP-1 cells was carried out by indirect immunofluorescence.

### RNA isolation and RT-PCR.

THP-1 cells grown at a density of 2 × 10^6^, either untransfected or transfected with pC-37FL, pC-37N, and pC-37C, were subjected to RNA isolation using TRIzol reagent (Promega) as per the manufacturer’s protocol. Reverse transcription was carried out with 2 µg RNA using the Promega RT kit after initial treatment with Promega RQ1 DNase as described by the manufacturer. The primers used for PCR are listed in Table S4 in the supplemental material.

10.1128/mBio.01712-17.8 TABLE S4  Primers used in this study. Download TABLE S4, DOCX file, 0.1 MB. Copyright © 2018 Ahmad et al. 2018 Ahmad et al. This content is distributed under the terms of the Creative Commons Attribution 4.0 International license.

### Immunofluorescence.

THP-1 cells seeded at 60% confluence (2 × 10^4^ cells/well) in 4-chamber slides were transfected with constructs PPE37FL, PPE37N, and PPE37C or incubated with rP37N or rM40N as described above. The cells were incubated at 37°C for 24 h posttransfection or incubated with the recombinant proteins for 2, 12, and 24 h. Immunostaining with anti-P37N antibody was performed as discussed elsewhere (35). The cross-reactivity of anti-P37N to rM40N was checked by Western blotting as well as immunoprecipitation. Immunofluorescence was visualized under a Zeiss LSM10 multiphoton fluorescence microscope. Images were processed using Adobe Photoshop 7 software.

### Immunoblotting.

Western blotting was carried out to examine the intracellular levels of Akt, IKKα, and caspase-3 in cell lysates and nuclear localization of NF-κB in nuclear extracts prepared from various groups. Forty micrograms of the cell lysates or 20 µg of nuclear lysate fractionated on 10% Tris-Tricine gel was used for immunoblotting, which was carried out as described earlier (34).

### Morphological determination of cells.

Phase-contrast microscopy was carried out to ascertain the morphological differences after transfection of the THP-1 cells with constructs or incubation with the recombinant protein to monitor cell proliferation, differentiation, and apoptosis of the THP-1 cells.

### Immunization of mice.

The study was conducted at an animal house facility at the National Institute of Pathology and was carried out strictly in accordance with the guidelines for animal handling of the institute. The animal ethics committee of the institute approved the protocol.

Eight- to 12-week-old C57BL/6J mice were procured from the National Institute of Immunology, New Delhi, India. The animals were placed at 5 to 6 females per group. The cages were placed in a room at a temperature of 24 ± 2°C, humidity of 45 to 50%, and an artificial photoperiod of 12-h day/12-h night. A period of 7 days was given for acclimatization to the environment and observation for signs of disease. Primary immunization was carried out with 30 μg/ml of recombinant PPE37FL and PPE37N proteins in PBS buffer, and control mice were injected with PBS buffer only subcutaneously at the base of the tail. Two booster doses were given subcutaneously with 30 μg/ml of recombinant PPE37FL and PPE37N protein at every 10-day interval. Finally, mice were sacrificed 10 days after the second booster dose.

### Sampling and sample preparation.

On day 30 post-primary immunization, animals were euthanized and blood was collected. Blood samples were stored in tubes without anticoagulant to obtain blood serum with natural clotting. The serum samples were frozen in an Eppendorf tube and stored at −80°C.

### Splenocyte isolation and single-cell preparation.

Spleens isolated from euthanized mice were crushed and perfused, and the cell suspension was transferred to another tube after passing through a cell strainer. Cells were centrifuged, washed, and resuspended in red blood cell (RBC) lysis buffer. The cells were centrifuged and finally resuspended in DMEM containing 10% fetal bovine serum (FBS). The humoral immune response was assayed by enzyme-linked immunosorbent assay (ELISA) of the antibody content in blood serum, and the cell-mediated immune response was assayed by flow cytometric analysis of splenocytes.

### Intracellular cytokine staining and extracellular staining of surface markers.

For cell-mediated immune response, splenocytes (1 × 10^6^/well) were seeded into a 96-well plate and restimulated with 3- and 5-μg/ml concentrations of recombinant PPE37FL or PPE37N for 12 h in the presence of monensin (BD) GolgiPlug and GolgiStop. Cells were collected, washed with PBS, and stained with anti-CD4 and anti-CD8 antibodies (CD3a-fluorescein isothiocyanate [FITC], CD4-allophycocyanin [APC]-H7, and CD8-peridinin chlorophyll protein [PerCP]-Cy5.5). Cells were fixed with 4% paraformaldehyde, permeabilized in 0.02% Triton X-100 followed by washing, and stained with anti-IFN-γ and anti-TNF-α (APC for IFN-γ and phycoerythrin [PE]-Cy7 for TNF-α) for 1 h.

### Immune assays.

Experiments involving human patients were approved by the Institutional Ethics Committee. Humoral immune response from serum samples was assayed by ELISA. To assess the humoral immunity, Corning plates were sensitized with 10-μg/ml solutions of the recombinant antigens PPE37FL and PPE37N as described earlier (41). Briefly, 96-well plates were coated with specific proteins (10 μg/ml) in coating buffer and kept at 4°C overnight. Plates were washed three times with wash buffer and blocked for 2 h at room temperature. After three washes, serum samples in 1:200 dilutions were added and kept for 2 h. Plates were again washed 4 times. Secondary conjugate antibody (horseradish peroxidase [HRP] conjugated, 1:10,000 dilution) was added for 2 h. The plate was washed at least five times, *o*-phenylenediamine dihydrochloride (OPD) substrate was added, and the reaction was stopped with 3 N H_2_SO_4_.

Cytokine secretion induced by PPE37FL and PPE37N antigens in splenocytes was also quantified using ELISA as described earlier (35). Splenocytes were restimulated with 3 μg/ml and 5 μg/ml PPE37FL and PPE37N antigens for 48 and 96 h. Supernatants were harvested after 48 and 96 h and quantified for various cytokine levels. Briefly, 96-well ELISA plates were coated with capture antibody in coating buffer (bicarbonate/phosphate buffer) kept at 4°C overnight. Plates were washed with PBS-T (PBS plus 0.05% Tween 20) three times. Five percent bovine serum albumin (BSA) was used as blocking buffer as well as assay diluent. After 2 h of blocking, the collected supernatant along with standards was added for 2 h. After 5 washes, detection antibody and enzyme conjugates were added for 1 h. After 7 washes, ABTS [2,2′-azinobis(3-ethylthiazolinesulfonic acid)] substrate was added, and 3 N H_2_SO_4_ was added to stop the reaction. Absorbance was measured at 450 nm, and a standard curve was plotted along with standards to determine the cytokine levels in test samples.

### Apoptosis assay in the presence of inhibitor.

HEK293T cells were seeded into 6-well plates. Transfection was carried out with plasmids containing full-length N- and C-terminal segments of PPE37 and pcDNA3.1 as the control vector. Cells transfected with pC-PPE37C were also incubated with apoptosis inhibitor SR-VAD-FMK (a sulforhodamine-labeled fluoromethyl ketone peptide inhibitor of caspase [Guava Technologies]). Cells were collected after 24 h of incubation, and apoptosis was assayed using an FITC-annexin V apoptosis detection kit with 7-aminoactinomycin D (7-AAD [BioLegend]). Briefly, cells were washed twice with BioLegend’s cell staining buffer and then resuspended in 7-AAD–annexin V binding buffer at a concentration of 1 × 10^7^cells/ml, followed by transfer of 100 μl of cell suspension in a 5-ml test tube. To this was added 5 μl of FITC-annexin V4 and 5 μl of 7-AAD viability staining solution. Cells were gently vortexed and incubated for 15 min at room temperature (25°C) in the dark. To each tube was added 400 μl of annexin V binding buffer, and the mixture was analyzed by flow cytometry with proper machine settings.

### Statistical analysis.

Data are expressed as the mean ± standard deviation (SD) from three independent experiments performed with similar results. Analysis was carried out using Student’s *t* test wherever applicable. *P* < 0.05 was considered to be significant.
