# Supplementary material for: Contrasting Function of Structured N-Terminal and Unstructured C-Terminal Segments of Mycobacterium tuberculosis PPE37 Protein
Source: mBio. 2018 Jan 23;9(1):e01712-17. doi: 10.1128/mBio.01712-17 (PMC5784249; doi:10.1128/mBio.01712-17)
Supplement: TABLE S1 [file mbo006173677st1.docx]

**Table S1: *Mycobacterium* tuberculosis strains used for comparative analysis.**

| **Strain Information** | **Acession Number** |
| --- | --- |
| Mycobacterim tuberculosis F11 | CP000717.1 |
| Mycobacterim tuberculosis CDC 1551 | AE000516.2 |
| Mycobacterium tuberculosis CCDC5079 | CP002884.1 |
| Mycobacterium tuberculosis CCDC5180 | CP001642.1 |
| Mycobacterium tuberculosis KZN 605 | CP001976.1 |
| Mycobacterium tuberculosis KZN 1435 | CP001658.1 |
| Mycobacterium tuberculosis KZN 4207 | CP001662.1 |
| Mycobacterium tuberculosis str. Haarlem | CP001664.1 |
| Mycobacterium tuberculosis str. Beijing/NITR203 | CP005082.1 |
| Mycobacterium tuberculosis EAI5/NITR206 | CP005387.1 |
| Mycobacterium tuberculosis 49-02 | HG813240.1 |
| Mycobacterium tuberculosis 7199-99 | HE663067.1 |
| Mycobacterium tuberculosis BT1 | CP002883.1 |
| Mycobacterium tuberculosis BT2 | CP002882.1 |
| Mycobacterium tuberculosis CTRI-2 | CP002992.1 |
| Mycobacterium tuberculosis EAI5 | CP006578.1 |
| Mycobacterium tuberculosis H37RvSiena | CP007027.1 |
| Mycobacterium tuberculosis HKBS1 | CP002871.1 |
| Mycobacterium tuberculosis K | CP007803.1 |
| Mycobacterium tuberculosis W-148 | CP012090.1 |
| Mycobacterium tuberculosis str. Erdman = ATCC 35801 | AP012340.1 |
| Mycobacterium tuberculosis str. Kurono DNA | AP014573.1 |
| Mycobacterium tuberculosis strain 96075 | CP009426.1 |
| Mycobacterium tuberculosis strain 96121 | CP009427.1 |
| Mycobacterium tuberculosis strain KIT87190 | CP007809.1 |
| Mycobacterium tuberculosis strain SCAID 187.0 | CP012506.1 |
| Mycobacterium tuberculosis strain ZMC13-88 | CP009101.1 |
| Mycobacterium tuberculosis strain ZMC13-264 | CP009100.1 |
