# Supplementary material for: Contrasting Function of Structured N-Terminal and Unstructured C-Terminal Segments of Mycobacterium tuberculosis PPE37 Protein
Source: mBio. 2018 Jan 23;9(1):e01712-17. doi: 10.1128/mBio.01712-17 (PMC5784249; doi:10.1128/mBio.01712-17)
Supplement: TABLE S2 [file mbo006173677st2.docx]

**Table S2: *Mycobacterium* species used for comparative analysis.**

| MycobacterimAbscessus ATCC 19977 |
| --- |
| Mycobacterim sp. MCSN |
| Mycobacterium Avium subsp. Paratuberculosis k-10 |
| Mycobacterium Africanum GM041182 |
| Mycobacterium bovis BCG str. Pasteur 1173P2 |
| Mycobacterium cannettii CIPT-140010059 |
| Mycobacterium intracellulare ATCC 13950 |
| Mycobacterium kansaii ATCC 12478 |
| Mycobacterium marinum |
| Mycobacterium smegmatis str. MC2 |
| Mycobacterium ulcerans Agy99 |
| Mycobacterium chubuense NBB4 |
| Mycobacterium fortuitum CT6 |
| Mycobacterium indicuspranii |
| Mycobacterium leprae |
| Mycobacterium neoaurumvkc ac-1815d |
| Mycobacterium rhodesiae NBB3 |
| Mycobacterium chelonae |
| Mycobacterium gilvumpyrgck |
| Mycobacterium sinsense |
| Mycobacterium vanbaaleni |
| Mycobacterium goodie |
| Mycobacterium gilvum spy1 |
| Mycobacterium abscessus subspecies bolletii |
