# Supplementary material for: Contrasting Function of Structured N-Terminal and Unstructured C-Terminal Segments of Mycobacterium tuberculosis PPE37 Protein
Source: mBio. 2018 Jan 23;9(1):e01712-17. doi: 10.1128/mBio.01712-17 (PMC5784249; doi:10.1128/mBio.01712-17)
Supplement: FIG S1 [file mbo006173677sf1.docx]

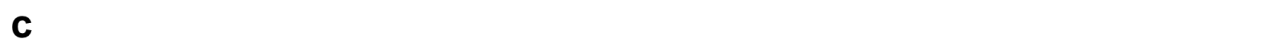
**
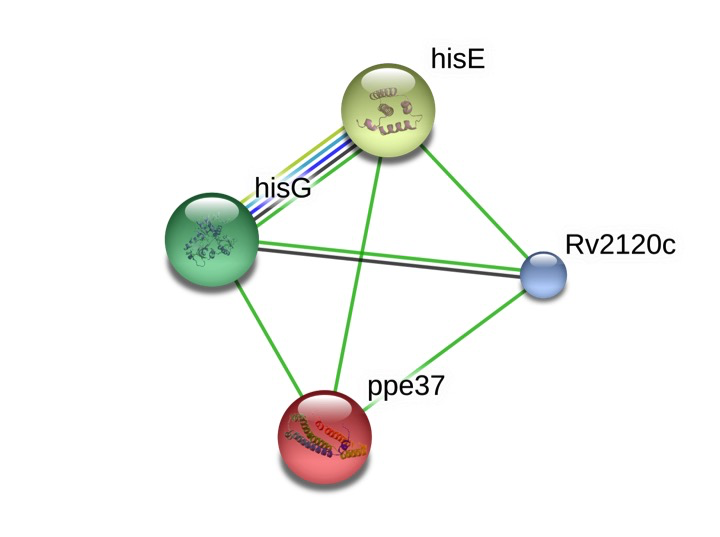
**

**Figure S1:** Sequence similarities between PPE37 and other members of PE PPE family.

**(a)** Multiple sequence alignment of representative orthologs of PPE37 by Clustal Omega with likely functional interaction motifs named and highlighted in different colors. The NCBI identifiers of the protein sequences are provided following the species names in the first line of the alignment. Motifs were searched by the ELM browser in each sequence and highlighted even if positionally not fitted with the corresponding H_37_Rv PPE37 motif. **(b)** Multiple sequence alignment of the five PPE37 paralogs by Clustal Omega with likely functional interaction motifs are named and highlighted in different colors. Due to the poor conservation of sequences in certain regions, the motifs were searched by ELM browser in each sequence and highlighted even if not fitted with the respective PPE37 motif. Three strongly conserved motif-like regions of unknown function, at the C-terminal, are marked by blue rectangles. Clustal consensus patterns are indicated below the sequences as in Figure 1 panel c.**(c)**STRING analysis of PPE37 for Protein-protein interacting partners which include hisE (phosphoribosyl-ATP pyrophosphatase*), hisG (*ATP phosphoribosyltransferase) and hypothetical protein Rv2120c.
