# Supplementary material for: Contrasting Function of Structured N-Terminal and Unstructured C-Terminal Segments of Mycobacterium tuberculosis PPE37 Protein
Source: mBio. 2018 Jan 23;9(1):e01712-17. doi: 10.1128/mBio.01712-17 (PMC5784249; doi:10.1128/mBio.01712-17)
Supplement: FIG S2 [file mbo006173677sf2.docx]

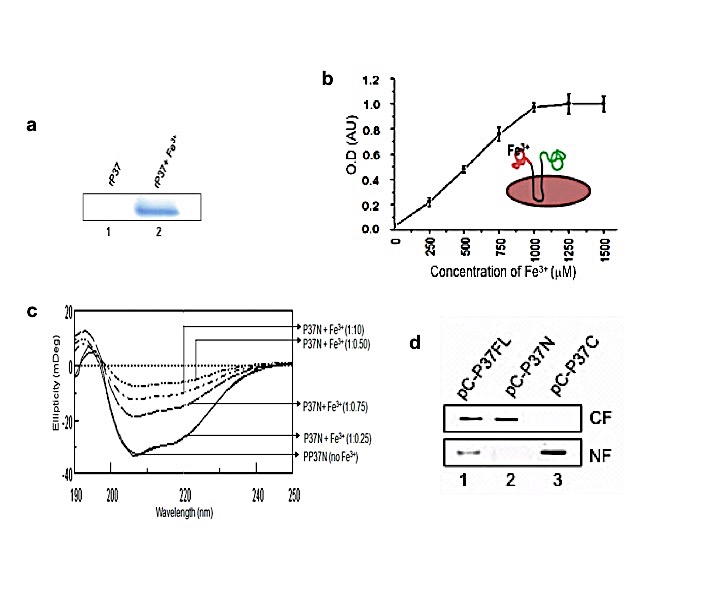


**Figure S2:** Recombinant PPE37 protein binds iron**. (a)** Ferrene-s staining of rPPE37 (lanes 1,2) was carried out in the absence (lane 1) or presence (lane 2) of iron. **(b)** Atomic absorption spectroscopy revealed iron-binding property of PPE37 as a direct function of iron concentration. **(c**) Circular dichroism spectrum of rPPE37N (N-terminal segment) at different concentration of Ferrous iron. **(d)** Immunoblot confirms localization of N-terminal and C-terminal to cytoplasm and nucleus, respectively. Cytoplasmic and nuclear lysates prepared from THP-1 cells transfected with pC-P37FL (lane 1), or pC-P37N (lane 2) or pC-P37C (lane 3) were immunoblotted using anti-P37FL antibody.
