# Supplementary material for: Contrasting Function of Structured N-Terminal and Unstructured C-Terminal Segments of Mycobacterium tuberculosis PPE37 Protein
Source: mBio. 2018 Jan 23;9(1):e01712-17. doi: 10.1128/mBio.01712-17 (PMC5784249; doi:10.1128/mBio.01712-17)
Supplement: FIG S3 [file mbo006173677sf3.docx]

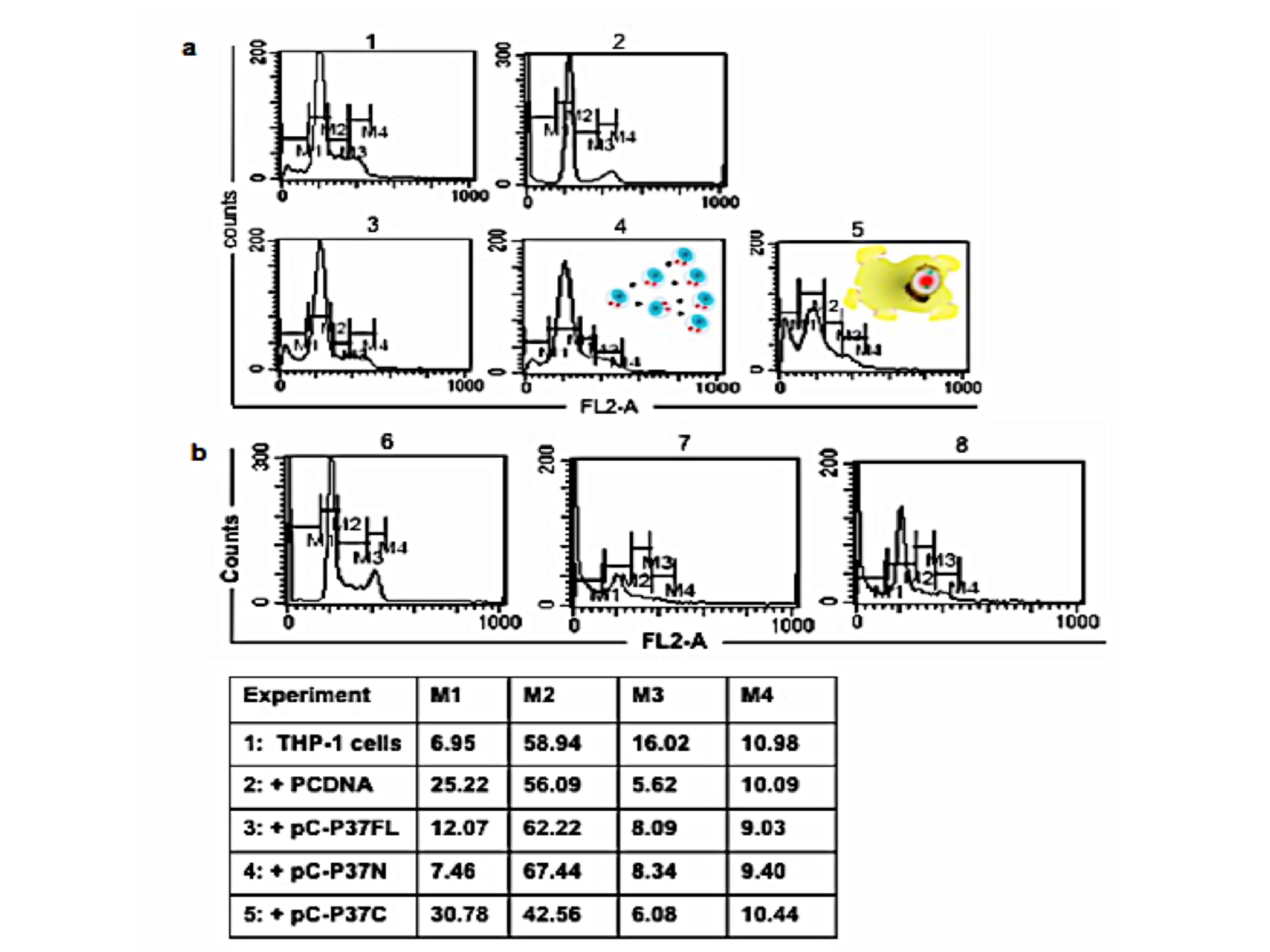


**Figure S3:** Cell cycle analyses of THP1 cells transfected with various constructs.

**(a)** Histograms indicating percentage of cells in each cell cycle phase for THP-1 cells, THP-1 cells transfected with pcDNA 3.1, or pC-P37FL, or pC-P37N or pC-P37C after 24 hours of transfection. pC-P37N transfected cells (4) showed an increase in the S phases whereas pC-P37C transfected cells (5) showed higher G_0_ phase indicating cell death. The quantitative estimation of cells at different stages of cell cycle is shown in the box on the right. **(b)** Cell cycle profile of THP-1 cells (6) at the start of 2’2’-dipyridyl treatment, t=0h; (7) after 6 hours of treatment followed by 18 hours of recovery in RPMI, t=24h and (8) after incubation with rP37N in the presence of 2’2’-dipyridyl, the treatment was carried out for 6 hours followed by recovery in RPMI, t=24h.
