# Supplementary material for: Contrasting Function of Structured N-Terminal and Unstructured C-Terminal Segments of Mycobacterium tuberculosis PPE37 Protein
Source: mBio. 2018 Jan 23;9(1):e01712-17. doi: 10.1128/mBio.01712-17 (PMC5784249; doi:10.1128/mBio.01712-17)
Supplement: FIG S4 [file mbo006173677sf4.docx]

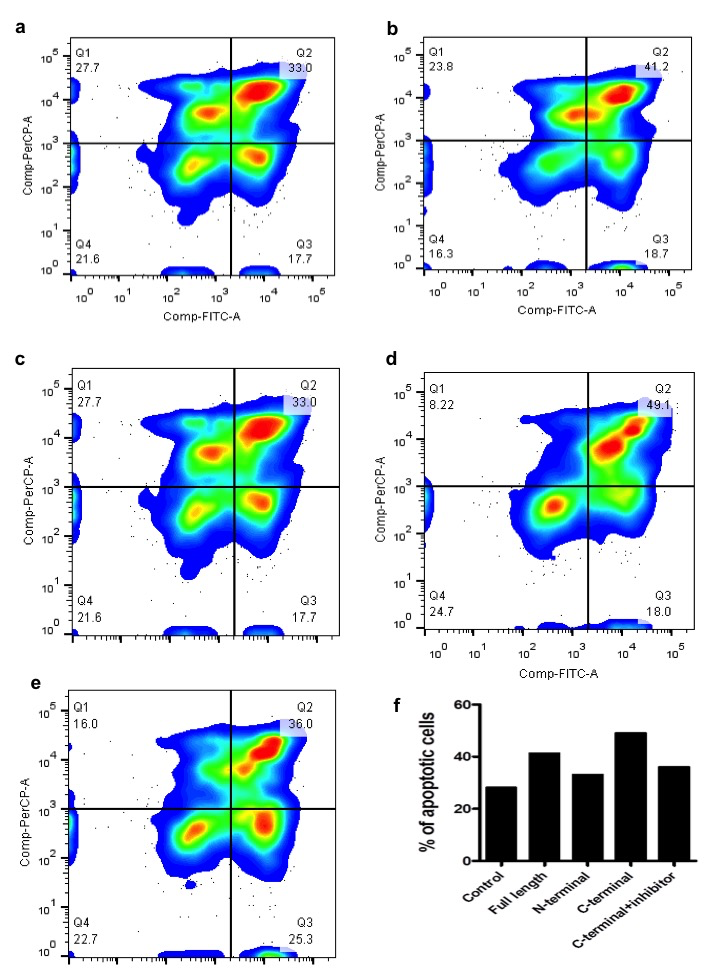


**Figure S4:** Apoptosis assay in presence of apoptotic inhibitor:

pcDNA constructs of PPE37FL, PPE37N and PPE37C were transfected in HEK-293T cells and apoptosis analysis was done using flow cytometric procedure. Representative FACS plots are shown in (Fig. S4) (a) pcDNA3.1 (+) control. (b) pC-PPE37FL, (c) pC-PPE37N, (d) pC-PPE37C, (e) pC-PPE37C +inhibitor. (f) Number of apoptotic cells is represented by bar diagram.
