# Supplementary material for: Contrasting Function of Structured N-Terminal and Unstructured C-Terminal Segments of Mycobacterium tuberculosis PPE37 Protein
Source: mBio. 2018 Jan 23;9(1):e01712-17. doi: 10.1128/mBio.01712-17 (PMC5784249; doi:10.1128/mBio.01712-17)
Supplement: TABLE S3 [file mbo006173677st3.docx]

**Table S3: Protein Families and secretome used in analysis.**

PE subfamily

| Gene Id | Protein name | Gene Id | Protein name | Gene Id | Protein name |
| --- | --- | --- | --- | --- | --- |
| YP_177690.1 | PE1 | YP_177794.1 | PE13 | YP_177882.1 | PE25 |
| YP_177696.1 | PE2 | YP_177797.1 | PE14 | YP_177907.1 | PE27 |
| YP_177697.1 | PE3 | YP_177805.1 | PE15 | YP_177685.1 | PE29 |
| YP_177710.1 | PE5 | YP_177810.1 | PE16 | YP_177975.1 | PE31 |
| YP_177717.1 | PE6 | YP_177825.1 | PE17 | YP_177999.1 | PE32 |
| YP_177766.1 | PE7 | YP_177834.1 | PE18 | YP_178000.1 | PE33 |
| YP_177779.1 | PE8 | YP_177837.1 | PE19 |  |  |
| YP_177784.1 | PE9 | YP_177843.1 | PE20 |  |  |
| CCP43841.1 | PE10 | YP_177858.1 | PE22 |  |  |
| YP_177792.1 | PE11/LIPX | YP_177867.1 | PE23 |  |  |
| YP_177793.1 | PE12 | YP_177880.2 | PE24 |  |  |

PPE subfamily

| Gene Id | Protein name | Gene Id | Protein name | Gene Id | Protein name |
| --- | --- | --- | --- | --- | --- |
| YP_177690.1 | PPE1 | YP_177830.1 | PPE24 | YP_177918.1 | PPE46 |
| YP_177704.1 | PPE2 | YP_177833.1 | PPE25 | YP_177932.1 | PPE49 |
| YP_177709.1 | PPE3 | YP_177835.1 | PPE26 | YP_177934.1 | PPE50 |
| YP_177711.1 | PPE4 | YP_177836.1 | PPE27 | YP_177935.1 | PPE51 |
| YP_177714.1 | PPE5 | YP_177839.1 | PPE28 | YP_177936.2 | PPE52 |
| YP_177715.1 | PPE6 | YP_177840_1 | PPE29 | YP_177937.1 | PPE53 |
| YP_177720.1 | PPE7 | YP_177841.1 | PPE30 | YP_177960.1 | PPE54 |
| YP_177721.2 | PPE8 | CCP44573.1 | PPE31 | YP_177963.1 | PPE55 |
| YP_177726.2 | PPE10 | YP_177844.1 | PPE32 | YP_177964.1 | PPE56 |
| YP_177727.1 | PPE11 | YP_177845.1 | PPE33 | YP_177971.1 | PPE57 |
| YP_177753.1 | PPE12 | YP_177655.1 | PPE34 | YP_177972.1 | PPE58 |
| YP_177764.1 | PPE13 | YP_177850.1 | PPE35 | YP_177973.1 | PPE59 |
| YP_177765.1 | PPE14 | YP_177859.1 | PPE36 | YP_177976.1 | PPE60 |
| YP_177778.1 | PPE15 | YP_177861.1 | PPE37 | YP_177984.1 | PPE61 |
| YP_177790.1 | PPE16 | YP_177870.1 | PPE38 | YP_177985.1 | PPE62 |
| YP_177791.1 | PPE17 | YP_177871.1 | PPE39 | YP_177987.1 | PPE63 |
| YP_177795.1 | PPE18 | CCP45144.1 | PPE40 | YP_177988.1 | PPE64 |
| YP_177801.1 | PPE19 | YP_177881.1 | PPE41 | YP_177998.1 | PPE65 |
| YP_177806.1 | PPE20 | YP_177893.1 | PPE42 | YP_178009.1 | PPE66 |
| YP_177817.1 | PPE21 | YP_177906.1 | PPE43 | YP_178010.1 | PPE67 |
| YP_177827.1 | PPE22 | YP_177677.1 | PPE44 | YP_178022.1 | PPE68 |
| YP_177828.1 | PPE23 | YP_177913.1 | PPE45 | YP_178024.1 | PPE69 |

PE_PGRS subfamily

| Gene Id | Protein Name | Gene Id | Protein Name | Gene Id | Protein Name |
| --- | --- | --- | --- | --- | --- |
| YP_177692.1 | PE_PGRS1 | YP_177786.1 | PE_PGRS22 | YP_177891.1 | PE_PGRS44 |
| YP_177693.1 | PE_PGRS2 | YP_177798.1 | PE_PGRS23 | YP_177895.1 | PE_PGRS45 |
| YP_177707.1 | Rv0278c | YP_177799.1 | PE_PGRS24 | YP_177896.1 | PE_PGRS46 |
| YP_177708.1 | PE_PGRS4 | YP_177809.1 | PE_PGRS25 | YP_177902.1 | PE_PGRS47 |
| YP_177713.1 | PE_PGRS5 | YP_177811.1 | PE_PGRS26 | YP_177909.1 | PE_PGRS48 |
| YP_177736.1 | PE_PGRS6 | YP_177812.1 | PE_PGRS27 | YP_177961.2 | PE_PGRS49 |
| YP_177739.1 | PE_PGRS7 | YP_177813.1 | PE_PGRS28 | YP_177962.1 | PE_PGRS50 |
| YP_177749.1 | PE_PGRS8 | YP_177814.1 | PE_PGRS29 | YP_177965.1 | PE_PGRS51 |
| YP_177750.1 | PE_PGRS9 | YP_177826.1 | PE_PGRS30 | YP_177968.1 | PE_PGRS52 |
| YP_177751.1 | PE_PGRS10 | YP_177832.1 | PE_PGRS31 | YP_177978.1 | PE_PGRS53 |
| YP_177752.1 | PE_PGRS11 | YP_177842.1 | PE_PGRS32 | YP_177979.1 | PE_PGRS54 |
| YP_177759.1 | PE_PGRS12 | YP_177846.1 | PE_PGRS33 | YP_177980.1 | PE_PGRS55 |
| YP_177760.1 | PE_PGRS13 | YP_177847.1 | PE_PGRS34 | YP_177981.2 | PE_PEGRS56 |
| YP_177761.1 | PE_PGRS14 | YP_177854.1 | PE_PGRS35 | YP_177982.1 | PE_PGRS57 |
| YP_177763.1 | PE_PGRS15 | YP_177862.1 | PE_PGRS37 | YP_177993.1 | PE_PGRS58 |
| YP_177773.1 | PE_PGRS16 | YP_177865.1 | PE_PGRS38 | YP_177994.1 | PE_PGRS59 |
| YP_177774.1 | PE_PGRS17 | YP_177869.1 | PE_PGRS39 | YP_178001.1 | PE_PGRS60 |
| YP_177775.1 | PE_PGRS18 | YP_177875.1 | PE_PGRS40 | YP_178002_1 | PE_PGRS61 |
| YP_177780.1 | PE_PGRS19 | YP_177878.1 | PE_PGRS41 | YP_178019_1 | PE_PGRS62 |
| YP_177781.1 | PE_PGRS20 | YP_177886.1 | PE_PGRS42 | YP_177924.1 | PE_PGRS63 |
| YP_177783.1 | PE_PGRS21 | YP_177887.1 | PE_PGRS43 |  |  |
