# Supplementary material for: Contrasting Function of Structured N-Terminal and Unstructured C-Terminal Segments of Mycobacterium tuberculosis PPE37 Protein
Source: mBio. 2018 Jan 23;9(1):e01712-17. doi: 10.1128/mBio.01712-17 (PMC5784249; doi:10.1128/mBio.01712-17)
Supplement: TABLE S4 [file mbo006173677st4.docx]

**Table S4: Primers used in this study**.

| Rv213FLFSacI | AAGAGCTCGTGACCTTCCCGATGTGG |
| --- | --- |
| Rv2123FLRHindIII | ATAAGCTTATCTGACCCGTCCCACGTGT |
| Rv2123NtRHindIII | ATAAGCTTGGCGATGAGGTCGCCGAGCG |
| Rv2123CTFSacI | AAGAGCTCG CCGGACCAATGGTCGAAC |
| Rv2123FLFpHdIII | TTAAGCTTGTAATGGTGACCTTCCCGATGTGGTT |
| Rv2123FLRpXhoI | ATCTCGAG TTAATCTGACCCGTCCCACGT |
| Rv2123CTDFpHdIII | TTAAGCTTGTAATG GCCGGACCAATGGTCGA |
| Rv2123NTDRpXhoI | ATCTCGAGTTAGCGGCCCCGGCAGCAGC |
